# Mesothelin confers pancreatic cancer cell resistance to TNF-α-induced apoptosis through Akt/PI3K/NF-κB activation and IL-6/Mcl-1 overexpression

**DOI:** 10.1186/1476-4598-10-106

**Published:** 2011-08-31

**Authors:** Uddalak Bharadwaj, Christian Marin-Muller, Min Li, Changyi Chen, Qizhi Yao

**Affiliations:** 1 Michael E. DeBakey Department of Surgery, Molecular Surgeon Research Center, Baylor College of Medicine, Houston, TX 77030, USA; 2 Department of Molecular Virology and Microbiology, Baylor College of Medicine, Houston, Texas 77030, USA; 3 The Vivian L. Smith Department of Neurosurgery, The University of Texas Health Science Center at Houston, Medical School, Houston, TX 77030, USA

**Keywords:** Pancreatic cancer, Mesothelin, TNF-α, Apoptosis

## Abstract

**Background:**

Previous studies showed that mesothelin (MSLN) plays important roles in survival of pancreatic cancer (PC) cells under anchorage dependent/independent conditions as well as resistance to chemotherapy. The recent success of intratumorally-injected adeno-encoded, chemo/radiation-inducible-promoter driven hTNF-α, (TNFerade) + gemcitabine in pre-clinical models of PC have renewed interest in use of TNF-α as a therapeutic component. To help find additional factors which might affect the therapy, we examined the resistance of MSLN-overexpressing pancreatic cancer cell lines to TNF-α-induced growth inhibition/apoptosis.

**Methods:**

Stable MSLN overexpressing MIA PaCa-2 cells (MIA-MSLN), stable MSLN-silenced AsPC-1 cells (AsPC-shMSLN) and other pancreatic cells (MIA-PaCa2, Panc 28, Capan-1, BxPC3, PL 45, Hs 766T, AsPC-1, Capan-2, Panc 48) were used. NF-κB activation was examined by western blots and luciferase reporter assay. TNF-α induced growth inhibition/apoptosis was measured by MTT, TUNEL assay and caspase activation. IL-6 was measured using luminex based assay.

**Results:**

Compared to low endogenous MSLN-expressing MIA PaCa-2 and Panc 28 cells, high endogenous MSLN-expressing Capan-1, BxPC3, PL 45, Hs 766T, AsPC-1, Capan-2, Panc 48 cells were resistant to TNF-α induced growth inhibition. Stable MSLN overexpressing MIA-PaCa2 cells (MIA-MSLN) were resistant to TNF-α-induced apoptosis while stable MSLN-silenced AsPC1 cells (AsPC-shMSLN) were sensitive. Interestingly, TNF-α-treated MIA-MSLN cells showed increased cell cycle progression and cyclin A induction, both of which were reversed by caspase inhibition. We further found that MIA-MSLN cells showed increased expression of anti-apoptotic Bcl-XL and Mcl-1; deactivated (p-Ser^75^) BAD, and activated (p-Ser^70^) Bcl-2. Constitutively activated NF-κB and Akt were evident in MIA-MSLN cells that could be suppressed by MSLN siRNA with a resultant increase in sensitivity of TNF-α induced apoptosis. Blocking NF-κB using IKK inhibitor wedelolactone also increased sensitivity to TNF-α-mediated cytotoxicity with concomitant decrease in Mcl-1. Blocking Akt using PI3K inhibitor also had a likewise effect presumably affecting cell cycle. MIA-MSLN cells produced increased IL-6 and were increased furthermore by TNF-α treatment. SiRNA-silencing of IL-6 increased TNF-α sensitivity of MIA-MSLN cells.

**Conclusions:**

Our study delineates a MSLN-Akt-NF-κB-IL-6-Mcl-1 survival axis that may be operative in PC cells, and might help cancer cells' survival in the highly inflammatory milieu evident in PC. Further, for the success of TNFerade + gemcitabine to be successful, we feel the simultaneous inhibition of components of this axis is also essential.

## Background

The importance of mesothelin (MSLN) as a biomarker and preferred immunotherapeutic target is steadily growing for many cancers, including pancreatic cancer (PC) [1-3]. The functional consequence of MSLN overexpression in various neoplasms has only recently begun to emerge. Evidence suggests that MSLN confers resistance to anoikis in breast cancer [4] and chemotherapy (platinum + cyclophosphamide/paclitaxel) in ovarian cancer [5]. In pancreatic cancer, it was suggested that MSLN is up-regulated following K-RAS, p53, p16 mutations [6], denoting its advantage in surviving early genotoxic insult. Our previous data [1] showed that MSLN-induced Stat3/cyclin E promotes survival/proliferation of pancreatic cancer cells under reduced serum conditions. Studies to ascertain role of MSLN in resisting other kinds of stress/apoptotic stimuli are thus warranted.

Tumor necrosis factor-*α *(TNF-α) is a vital member of the TNF-α super family, and plays roles in immunity, cellular remodeling, apoptosis and cell survival [7]. It acts primarily through tumor necrosis factor receptor-1 (TNFR1) (55 kD) to induce apoptosis by activating caspases through both mitochondria-dependent and independent pathways. A second receptor, TNFR2 (75 kD), signals primarily in immune cells [8]. TNF-α was identified as a cytokine that induces tumor necrosis/regression in animals [9]. Early studies suggesting an increased TNF-α sensitivity in oncogene/chemically transformed cells [10,11] aroused huge hopes but eventually subsided because of the issue of systemic toxicity. Recently, intratumorally-injected adeno-encoded, chemo/radiation-inducible-promoter driven hTNF-α, (TNFerade) in conjunction with conventional chemotherapy (e.g. gemcitabine in pre-clinical models of PC without metastasis at diagnosis) is largely devoid of the toxicity issue and has generated renewed interest in TNF- α treatment [12,13]. However, a large percentage of patients and/or cell lines are resistant to TNF-α treatment [14,15]. TNF-α also plays a significant role in the inflammatory etiology of pancreatic cancer [16,17]. Macrophages and other immune cells invading the tumor space, and tumor cells themselves, secrete TNF-α [17,18]. TNF-α was found to support pancreatic cancer cell growth through epidermal growth factor receptor (EGFR) and transforming growth factor (TGF-α) expression [19]. Thus factors determining cell fate in presence of TNF-α need to be studied. Therefore, there is a need to: 1) Identify TNF-α-responsive cells to select patients potentially responsive to TNF-α; and 2) ascertain factors responsible for resistance in an effort to improve therapeutic approaches.

NF-κB proteins are transcription factors induced in response to inflammatory and other stress stimuli [17]. A majority of cancer cells become resistant to TNF-α as a result of the activation of NF-κB [17] and consequent induction of anti-apoptotic molecules (e.g. IAPs/Bcl-XL), as the pro-survival effects of TNF-α out-perform the pro-apoptotic effects. Literature shows that blocking NF-κB activation can overcome TNF-α resistance [17], although a constitutive NF-κB activation, rather than the inducible one, has been suggested to be more important [20].

Here, we aimed at deciphering how MSLN overexpression in pancreatic cancer cells affects sensitivity to TNF-α-induced apoptosis. A panel of stable MSLN-overexpressing and MSLN-shRNA silenced cells were treated with TNF- α and examined for the changes of relevant signaling pathways, pro- and anti-apoptotic molecules and cytokine production. Our results demonstrate that MSLN overexpression leads to constitutive NF-κB/Akt activation resulting in resistance to TNF-α mediated cytotoxicity, presumably through de-activation of BAD, activation of Bcl-2 and upregulation of Mcl-1, in an IL-6 dependent manner. Our study may indicate a very important survival axis of MSLN-expressing pancreatic cancer cells in midst of inflammatory cytokines abundant in this cancer.

## Methods

### Cell culture, chemicals, and antibodies

Human pancreatic cancer cell lines MIA PaCa-2, Panc 28, Capan-1, BxPC3, PL 45, Hs 766T, AsPC-1, Capan-2, Panc 48 were purchased from the American Type Culture Collection (ATCC, Rockville, MD). Puromycin, anti-β-actin antibody were purchased from Sigma (St. Louis, MO). Recombinant human TNF-α was from R&D Systems. Antibodies for p-IκB-α/IκB-α/Bcl-XL/Bcl-2/Mcl-1/BAX/BAD/phospho-BAD/IKK-α/goat anti-rabbit IgG-HRP/goat anti-mouse IgG-HRP are from Cell Signaling Technology Laboratories Inc (Beverly, MA); p65 antibody was from Santa Cruz Biotechnology (Santa Cruz, CA); and Lamin A antibody from Abcam (Cambridge, MA). MSLN overexpressing stable cells were selected in PC cells using retroviral cloning as described previously [3].

### Cell proliferation measurement by cell viability assay (MTT)

Two thousand cells were plated and serum starved for 24 h. Various treatments were added to the media and the culture was continued for 2/4/6 days. Proliferating capacity was measured by dividing the OD (590 nm) at a time point by OD at 0 day (day after plating cells). Percent viability was measured by dividing OD value of the treated cells by that for untreated cells multiplied by 100.

### TUNEL assay

Apoptosis was evaluated by measuring DNA fragmentation using the Apo-Direct assay kit (Pharmingen, San Diego, CA) for TUNEL (terminal deoxynucleotidyltransferase dUTP nick end labeling). Briefly, cells were serum starved for 24 h (with or without pre-treatments) followed by treatment with 0/10/20/50 ng/ml of TNF-α, for 24/48/72 h. At each time-point, cells were harvested, fixed in 1% Paraformaldehyde, stained in 300 μL of propidium iodine/RNase solution and analyzed by flow cytometry.

### Real-Time RT-PCR

MSLN, TNFR1, TNFR2, and GAPDH mRNA levels were analyzed by real-time RT-PCR using the SYBR Green supermix kit (Bio-Rad) using 40 cycles at 95°C for 20 sec and 60°C for 1 min. Target gene mRNA was normalized to GAPDH mRNA level. Relative mRNA level was presented as unit values of 2^[Ct_(GAPDH) _- Ct_(gene of interest)_], Ct = threshold cycle. Primer sequences for the genes are as follows; hMSLN sense: 5'-CTCAACCCAGATGCGTTCTCG-3', hMSLN antisense: 5'-AGGTCCACATTGGCCTTCGT-3', hTNFR1 sense: 5'CCTGGTCATTTTCTTTGGTC TTTG-3', hTNFR1 antisense: 5'-GGGTGAAGCCTGGAGTGG-3', hTNFR2 sense: CCAAGCACCTCCTTCCTG, hTNFR2 antisense: CACCACTCCTATTATTAGTAGA CC, hGAPDH sense: 5'-TGCACCACCAACTGCTTA GC-3', hGAPDH antisense: 5'-GGCATGGACTGTGGTCATGAG-3'.

### Immunoblot analysis

Cells were lysed with lysis buffer (Cell Signaling Technology, Beverly, MA) with phosphatase and protease inhibitors. Proteins were then resolved by SDS-PAGE, transferred to nitrocellulose membrane (Bio-Rad Laboratories, Hercules, CA) and detected using specific primary antibodies, appropriate HRP-conjugated secondary antibodies and ECL detection system (Amersham Biosciences, UK). The nuclear/cytoplasmic extracts were prepared using the N-PER nuclear and cytoplasmic extraction reagents (Pierce Biotechnology, Rockford, IL, U. S. A).

### Treatment with Wedelolactone

MIA-MSLN and MIA-V were treated with 0, 12.5 or 25 μM of the IKK inhibitor Wedelolactone (Calbiochem La Jolla, CA). Cells were collected after 24 h and whole cell and cytoplasmic/nuclear extracts were prepared similarly as previously described [1] and used for western blot to detect various proteins. For functional assays following 12 h Wedelolactone treatment, cells were washed thoroughly, and then treated with various concentrations of TNF-α and outcomes measured.

### NF-κB Reporter Assay

One day after plating, cells were co-transfected with 10 μg of pGL3-NF-κB-Firefly luciferase reporter plasmid and 0.1 μg of pGL3-β-Actin-Renilla luciferase as an internal control. 24 h post-transfection, cells were harvested in reporter lysis buffer and lysates were assayed for luciferase activities using a dual luciferase assay kit (Promega, Madison, WI). Luciferase activities were normalized by the ratio of firefly and *Renilla *luciferase activities. All experiments were carried out in duplicates.

### MSLN and IL-6 shRNA/siRNA transfections

For MSLN shRNA transfection experiments, plasmid encoding MSLN shRNA (TR311377, Origene, Rockville, MD) and 29-mer shRNA encoding non-effective expression plasmid against GFP (TR30003) were used. MIA-MSLN and MIA-V cells were transfected with MSLN/IL-6 specific siRNA using Lipofectamine 2000 (Invitrogen, CA, USA). Cells treated with transfection reagent alone (mock) or a control scrambled siRNA (for MSLN siRNA experiments) were used as negative controls. Cells/supernatants were collected 48 h post transfection for mRNA/proteins extraction for real time-PCR/protein detection and 72 h for apoptosis assays.

### Statistical Analysis

A two tailed student's t-test was used to compare the statistical difference between two groups. The results were expressed as the mean with SD. The differences were considered statistically significant when the p value was ≤ 0.05, although various levels were represented in the figures with appropriate symbols in the legends.

## Results

### High MSLN expressing pancreatic cancer cells are resistant to TNF-α-induced growth inhibition/apoptosis

**Figure 1:**
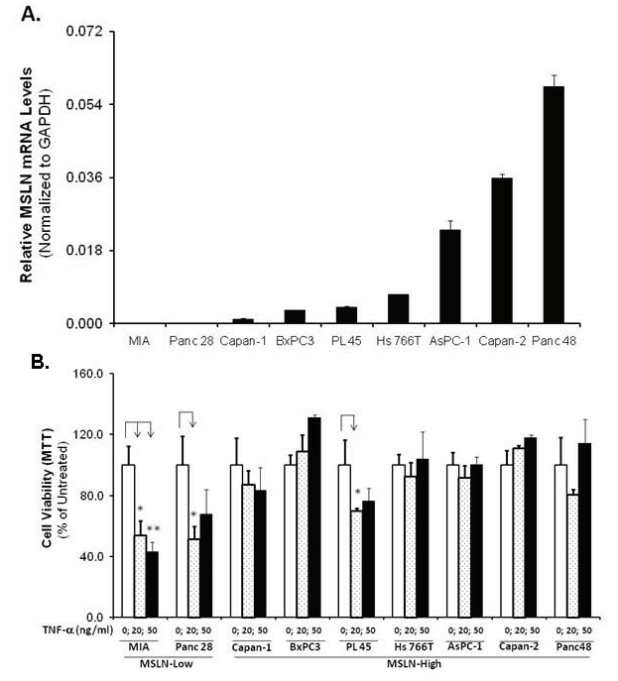
**MSLN expression level positively correlates with protection of pancreatic cancer cell lines from TNF-α induced apoptosis**. (A). Evaluation of MSLN mRNA expression in PC cell lines. Relative MSLN mRNA levels in pancreatic cancer cell lines MIA PaCa-2, Panc28, Capan-1, BxPC3, PL 45, Hs 766T, AsPC-1, Capan-2 and Panc 48 cells. Total mRNA from the cell lines were reverse transcribed and tested for MSLN expression by Real Time PCR. The results depicted denote MSLN mRNA levels in each cell line normalized to the GAPDH mRNA level. Relative mRNA level is presented as 2^[Ct(GAPDH)-Ct(MSLN)]. The bars denote SD of duplicate data. (B). A panel of PC cells' viability with or without TNF-α treatment. Cells were seeded in 96-well plates, serum-starved for 24 h, and then cultured in serum free medium ± 20/50 ng/ml of TNF-α for 72 h. Viable cells were quantitated by using MTT assay. Relative fold increase in viability is plotted along Y axis. Data plotted show means of triplicate wells.

To determine the role of MSLN in PC cells' sensitivity to TNF-α-induced growth-inhibition/apoptosis, cell lines with differential MSLN expression levels (Figure 1A) were used, including MSLN-low MIA PaCa-2 (MIA) [3] and Panc 28, and MSLN-high Capan-1, BxPC3, PL 45, Hs 766T, AsPC-1, Capan-2 and Panc 48 cells. Following serum starvation, cells were treated with 0/20/50 ng/ml of recombinant human TNF-α in serum free medium and the viability determined by MTT. As shown in Figure 1B, MSLN-high cells Capan-1, BxPC3, Hs 766T, AsPC-1, Capan-2 and Panc 48 were relatively resistant to growth inhibition by TNF-α, showing only about 8-17% reduction and in most cases these decreases were not significant. PL 45 was the only cell line which showed around 30% reduction in viability upon TNF-α treatment. At least three cell lines (BxPC3, Capan-2 and Panc 48) actually showed an increase in viability under identical conditions. In contrast, MSLN-low cells MIA PaCa-2 and Panc 28 had viability drastically reduced to 50-60% (*p < 0.05) upon TNF-α treatment. These data indicated a positive correlation between high MSLN expression and increased resistance against TNF-α. To test if serum level affects viability, two MSLN-high cells, BxPC-3/AsPC-1, were serum-starved and treated with 20 ng/ml of TNF-α in 0/0.2/2/10% FBS-containing media. As shown in Additional File 1, Fig. S1, TNF-α treatment did not affect viability in either BxPC-3 or AsPC-1 at any of these FBS concentrations. Given these results, we used serum free media for TNF-α treatment in subsequent experiments.

**Figure 2:**
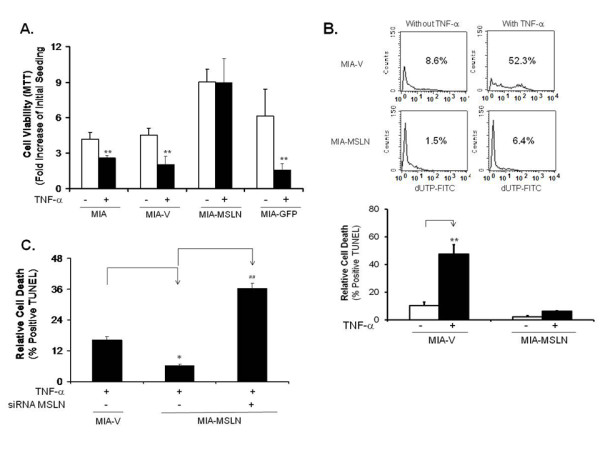
**MSLN overexpression protects PC cells from TNF-α induced cell viability decrease and apoptosis**. (A). MIA-MSLN and control cells viability assay with or without TNF-α treatment. About 3000 cells were plated in 96 well plates, serum starved for 24 h and treated with TNF-α at 20 ng/ml for 96 h after which viability was measured by MTT. Relative increase in viability was measured by dividing viability at a time point by viability of same cells at day 0 (day after plating) and is plotted along Y axis. Data plotted show mean of triplicate wells. Bars denote s.d. of triplicate data. *, # denote p < 0.05, **, ## denote p < 0.01, compared with controls by using student t test. (B). MIA-MSLN cells are resistant to TNF-α induced apoptosis using TUNEL assay. Cells were treated ± 20 ng/ml of TNF-α for 72 h and apoptosis was measured by TUNEL assay. Representative flow histograms show percentage of dUTP-FITC-positive apoptotic cells. Lower panel shows mean number of TUNEL positive cells (duplicate wells). (C). siRNA-silencing MSLN renders MIA-MSLN cells become TNF-α-sensitive measured by TUNEL assay. MIA-V/MIA-MSLN ells were plated in 6 well plates, transfected with siRNA, 24 h after transfection media was replaced by growth media, cells were serum starved and treated with TNF-α at 20 ng/ml for 72 h after which cells were collected, fixed and tested for DNA strand breaks by TUNEL assay. Bars denote s.d. of duplicate data. *, # denote p < 0.05, **, ## denote p < 0.01, compared with controls by using student t test.

To further confirm if MSLN overexpression in PC cells is responsible for resistance to TNF-α-induced apoptosis, we selected at least three different clones of MSLN stably overexpressed MIA PaCa-2 cell lines (MIA-MSLN) to study the role of MSLN in TNF-α-induced apoptosis. As shown in Figure 2A, the untransfected MIA PaCa-2 cells, the vector control cells (MIA-V), and the GFP expressing control cells (MIA-GFP) showed significant viability reduction (**p < 0.01) upon TNF-α treatment, but only MIA-MSLN cells were resistant to this effect (p > 0.05).

We then examined whether reduction in viability of control cells by TNF-α was due to decreased proliferation and/or increased apoptosis. As shown in Figure 2B about 50% of MIA-V cells underwent apoptosis after TNF-α treatment. Under similar conditions, only about 6% of MIA-MSLN cells underwent apoptosis. Bar chart (Figure 2B lower panel) from duplicate treatment wells showed minimal apoptosis in MIA-MSLN cells upon TNF-α treatment, compared to significant apoptosis in MIA-V cells (**p < 0.01)). The extent of apoptosis in MIA-V cells was time and TNF-α concentration-dependent (data not shown). To ascertain whether this protection against TNF-α was a direct effect of MSLN overexpression, we used specific siRNA against MSLN to knock down MSLN expression in MIA-MSLN cells and examine whether this could reverse the protective effect. As shown in Figure 2C, treatment with 10 ng/ml of TNF-α resulted in about 16% and 6% of MIA-V/mock-transfected MIA-MSLN cells undergoing apoptosis, respectively. However, significantly increased apoptosis (37%) was observed in MSLN-siRNA transfected cells. Additional File 2, Fig. S2 confirms that MSLN-specific siRNA significantly (p < 0.005) knocked down MSLN mRNA in MIA-MSLN cells. Furthermore, an examination of 7 high-MSLN-expressing cells Capan1, BxPC3, PL 45, AsPC-1, Capan-2, Panc 48 revealed that all of them were more resistant to TNF-α (20 ng/ml, 72 h) induced apoptosis (Table 1). When the base line TUNEL positivity (without TNF-α treatment) varied around 5%, TNF-α increased the percentages of TUNEL positivity to around 8.5% for most of them. AsPC-1 cells in fact showed a decrease in percentage of apoptotic cells from 4.7% to 1.8%. In comparison, MIA cells, with negligible MSLN expression were very sensitive and showed around 30% apoptosis (Table 1). These data suggest that MSLN overexpression has a direct effect on sensitivity/resistance of PC cells to TNF-α.

**Table 1:** Percent of cells undergoing apoptosis by TNF-α treatment

Cell Line	% TUNEL positive (without TNF-α)	% TUNEL positive (20 ng/ml TNF-α)
MIA	0.0	27.3

Capan-1	4.7	5.3

BxPC3	4.9	7.6

PL 45	4.5	2.9

AsPC-1	4.7	1.8

Capan-2	4.8	8.7

Panc 48	4.3	8.5

Cells were plated in 6 well plates, starved over night and treated with 20 ng/ml of TNF-α for 72 h. Cells were collected, fixed, washed and then processed for TUNEL assay.

### MSLN confers resistance to TNF-α-induced apoptosis through caspase-3 activation

**Figure 3:**
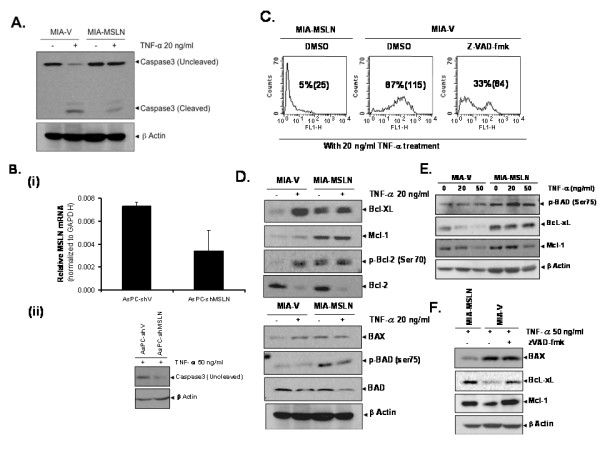
**MSLN overexpression makes PC cells resistant to TNF-α induced apoptosis through reduced Caspase 3 cleavage and up regulated anti-apoptotic proteins**. **(A)**. Reduced Caspase 3 cleavage in MIA-MSLN cells by 20 ng/ml of TNF-α treatment for 72 h. **(B)**. Silencing endogenous MSLN in AsPC-3 cells renders it sensitive to TNF-α induced apoptosis through increased Caspase 3 cleavage. Panel (i) shows the reduction of MSLN expression level in AsPC-shMSLN stable cell line. Panel (ii) shows the increased caspase 3 cleavage upon TNF-α treatment. **(C)**. Pre-treatment with pan-caspase inhibitor zVAD-fmk renders MIA-V cells resistant to TNF-α-induced apoptosis. Cells were treated with 20 ng/ml of TNF-α for 72 h and then stained with TUNEL assay. **(D)**. Pro and anti-apoptotic molecules expression in MIA-V/MIA-MSLN cells with or without treatment of 20 ng/ml TNF-α assayed by Western blot. **(E)**. Effect of different TNF-α doses on selected pro/anti-apoptotic molecules expression in MIA-V/MIA-MSLN cells. **(F)**. Effect of zVAD-fmk pre-treatment on various pro and anti-apoptotic molecules expression in MIA-V/MIA-MSLN cells after treatment with 50 ng/ml of TNF-α for 72 h.

To obtain further insight into the mechanism of TNF-α resistance in MIA-MSLN, we compared caspase-3 cleavage ± TNF-α treatment in MIA-V/MIA-MSLN cells. In accordance with our viability/TUNEL assay data, we found decreased caspase-3 cleavage in MIA-MSLN cells compared to MIA-V cells upon TNF-α treatment (Figure 3A). This result was confirmed in another PC cell line, AsPC-1. Shown in Figure 3B, silencing MSLN using MSLN specific shRNA, correlated with increased caspase-3 cleavage with TNF-α treatment. Furthermore, as shown in Figure 3C, when MIA-V cells were pre-treated with pan-caspase inhibitor zVAD-fmk, a reduced percentage of apoptosis (from 87% to about 33%) was observed, indicating that MIA-V cells undergo caspase-dependent apoptosis upon treatment with TNF-α, while MIA-MSLN cells are resistant.

### Upregulated anti-apoptotic proteins and downregulated pro-apoptotic proteins in MIA-MSLN cells

To further delineate the mechanism of TNF-α-resistance in MIA-MSLN cells, we examined expression of key anti/pro-apoptotic molecules ± TNF-α treatment. As shown in Figure 3D, two anti-apoptotic Bcl-2 family members, Bcl-XL/Mcl-1, were upregulated in MIA-MSLN cells. Importantly, Mcl-1 levels remained high even after TNF-α treatment. In MIA-V cells, on the other hand, Mcl-1 level increased slightly upon TNF-α treatment, but still remained lower than in MIA-MSLN cells. Another significant aspect is increased phosphorylation of anti-apoptotic molecule Bcl-2 at Ser70 position in its positive regulatory loop enhancing its anti-apoptotic action [21]. MIA-MSLN cells had increased p-Ser70; which could be a major factor behind the observed TNF-α resistance. In a dose-dependence study, we found that even with 50 ng/ml TNF-α treatment, Bcl-XL/Mcl-1 expression were still high in MIA-MSLN cells compared with control cells (Figure 3E). TNF-α treatment slightly decreased expression of two pro-apoptotic molecules, BAX and BAD (Figure 3D) in MIA-MSLN cells compared with a slight increase of BAX in control cells. Dephosphorylated BAD promotes apoptosis by binding and sequestering Bcl-2 and/or Bcl-XL away from pro-apoptotic Bax/Bak proteins [22,23]. Phosphorylated BAD, conversely, is bound and sequestered in cytosol by the chaperone protein 14-3-3. Phosphorylations at Ser75/Ser99/Ser118 are thus inhibitory for the pro-apoptotic function of BAD [24,25] as these affect interactions with 14-3-3 and/or Bcl-2 family members. The p-BAD (Ser75) level was consistently high in MIA-MSLN ± TNF-α treatment (Figure 3D, E) and thus might make BAD ineffective in inducing apoptosis in MIA-MSLN cells. Further, MIA-V pre-treatment with zVAD-fmk reversed the pattern of expression of the above pro- and anti-apoptotic molecules in the TNF-α treated MIA-V (Figure 3F), concomitant with the apoptosis reversal as shown in Figure 3C.

### TNF-α induced cyclin A and concomitant cell cycle progression in MIA-MSLN cells

**Figure 4:**
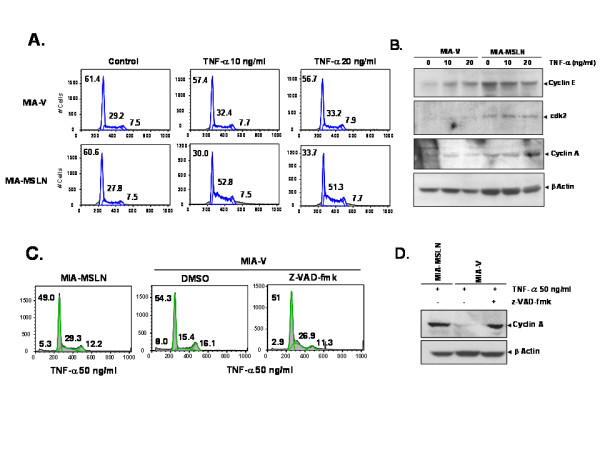
**TNF-α induces S phase progression and induction of cyclin A in MIA-MSLN cells**. **(A)**. Cell cycle analysis of MIA-V/MIA-MSLN cells treated with 10/20 ng/ml of TNF-α for 24 h. **(B)**. Cell cycle related molecules detection by western blot using the lysates from cells treated as in (A). **(C)**. Cell cycle analysis of MIA-V/MIA-MSLN cells after pre-treatment with zVAD-fmk and then treated with 50 ng/ml of TNF-α for 72 h. (D). Cyclin A detection by western blot using the cell lysates from cells treated as in (C).

Loss of cyclin A and G1-cell cycle arrest was found to precede cell-killing by TNF-α in endothelial cells [26]. TNF-α promoted cell cycle progression and cyclin A synthesis in serum starved HeLa cells to rescue them from pro-apoptotic effects [27]. To determine whether TNF-α treatment promotes cell cycle progression or arrest in MIA-MSLN cells, cell cycle analysis was used. Shown in Figure 4A, we found an induction of serum-starved MIA-MSLN cells into S phase (from 27.8% to 50%) by 10/20 ng/ml of TNF-α in presence of 0.2% serum with concomitant decrease of cells in G0/G1 phase. In contrast, the number of MIA-V cells going to S phase was much lower (from 29% to 33%). This indicates that TNF-a promotes growth of MSLN overexpressing cells. We further examined S-phase promoting cyclins A/E [28] and cdk2. Consistent with our previous report [1], cyclin E/cdk2 were slightly elevated in MIA-MSLN cells but remained unaffected by TNF-a treatment (Figure 4B). However, there was a clear induction of cyclin A in MIA-MSLN cells but not in MIA-V cells by TNF-α treatment.

Since we found previously that caspase inhibitor z-VAD-fmk rescued MIA-V cell from TNF-a-induced apoptosis, we examined its effect on both S phase progression and cyclin A induction. Interestingly, z-VAD-fmk pre-treatment not only decreased the apoptotic sub-G0 fraction (from 8% to 2.9%), it also increased MIA-V cells in S phase (from 15.4% to 26.9%) by TNF-α treatment (Figure 4C). This indicates that a failure to go into DNA synthesis is linked to MIA-V cells' induction to apoptosis by TNF-a treatment. In agreement with this observation, cyclin A expression also increased in z-VAD-fmk treated MIA-V cells (Figure 4D). Together, our data shows that TNF-a induces MSLN overexpressing PC cells progress to S phase through upregulation of cyclin A which makes the cells resistant its cytotoxic effects.

### The level of TNF receptor-1 expression is unchanged upon MSLN overexpression

To further dissect MIA-MSLN cells' resistance to TNF-α-induced apoptosis, we examined the expression of TNF receptors one (TNFR1) and two (TNFR2). Real-time PCR data showed there was high expression of TNFR1 (Additional File 3, Fig. S3A) and low expression of TNFR2 (data not shown) in both MIA-V and MIA-MSLN cells. TNFR1 was slightly decreased in MIA-MSLN cells (statistically insignificant). Western blot analysis showed that there was no significant difference of TNFR1 protein (~55 kD) expression between MIA-V and MIA-MSLN cells (Additional File 3, Fig. S3B). This data indicate that resistance of MSLN high expressing cells to TNF-α may not be caused by a differential receptor expression on these cells.

### MSLN overexpression induces NF-κB activation and nuclear translocation of its subunits in pancreatic cancer cell MIA PaCa-2

**Figure 5:**
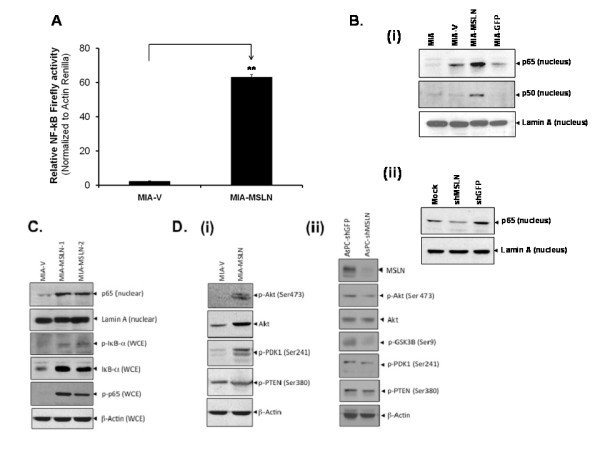
**MSLN overexpression is associated with activation of NF-κB and Akt activity in PC cells**. **(A)**. Enhanced NF-κB reporter gene activity in MIA-MSLN cells. Data plotted was measured as ratio of co-transfected firefly-NF-κB reporter normalized to Actin-Renilla. ** denote p < 0.01, compared with controls, t test. **(B)**. Nuclear protein from MIA-MSLN and control cells were subjected to western blot to detect nuclear-translocated p65, p50 and nuclear envelop protein Lamin A (panel i). Panel ii showed inhibited nuclear protein translocation when MIA-MSLN cells were transfected with MSLN specific shRNA encoding plasmid or control plasmids. **(C)**. Whole cell extract (WCE) or nuclear protein from MIA-V and two pools of MIA-MSLN cells were probed for various IκB-α degradation pathway proteins in western blot. **(D)**. Whole protein from MIA-V/MIA-MSLN (panel i) and from the AsPC-1 shMSLN and control cells (panel ii) were probed for various PI3k-Akt pathway proteins. Note: (panel i) data has individual lanes combined from a single electrophoresis gel.

To identify signals activated by MSLN, we examined various transcription factors, kinases, and related intermediates in MIA-MSLN cells. One of our major findings is that MIA-MSLN cells had significantly higher constitutive NF-κB activity than control cells. As represented in Figure 5A, relative NF-κB luciferase activity was increased 26-fold in MIA-MSLN cells compared to control cells. Figure 5B-(i) shows an increased nuclear translocation of p65/p50 subunits in MIA-MSLN cells. MSLN shRNA reduced nuclear translocation of p65 (Figure 5B, ii), indicating a causal relationship between MSLN overexpression and NF-κB activation.

Nuclear translocation of p65 was observed in two different MSLN overexpressing cell populations selected at different times (Figure 5C). We also observed an increased phosphorylation of NF-κB cytoplasm-sequestering inhibitor IκB-α (Figure 5C). Not surprisingly, we found an increased expression of IκB-α, a NF-κB regulated gene, in both MSLN overexpressing cells. An increased phosphorylation of NF-κB subunit p65 was also seen. All these data indicate that the IKK-IκB-α pathway may be operative in MSLN overexpressing cells.

### MSLN overexpression activates Akt activity in pancreatic cancer cells

To delineate MSLN-induced signaling, we also examined Akt pathway in MSLN overexpression or silencing stable cells. Shown in Figure 5D, we found activation of Akt (increased p-Thr473 and total Akt), the Akt kinase PDK1 (p-Ser241), and a deactivation of Akt inhibitor PTEN (p-Ser380) in MIA-MSLN cells. Correspondingly, the MSLN-silenced AsPC-1 cells (AsPC-shMSLN) had decreased p-Akt, p-GSK-3β (p-Ser9), PDK1 (p-Ser241) and PTEN (p-Ser380). Together, this data indicate that MSLN expression leads to Akt activation in PC cells.

### Abrogating the PI3K and NF-κB activity sensitizes MIA-MSLN cells to TNF-α-induced apoptosis

**Figure 6:**
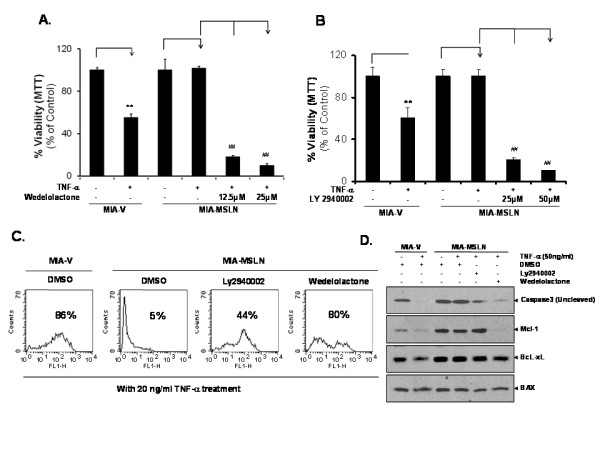
**Inhibiting NF-κB/Akt pathway or endogenous IL-6 abrogates MIA-MSLN cells resistance to TNF-α-induced apoptosis**. **(A)**. MIA-MSLN cell viability was reduced upon pre-treatment with IKK inhibitor wedelolactone and then 20 ng/ml of TNF-α for 72 h. **(B)**. MIA-MSLN cell viability was reduced upon pre-treatment with PI3K inhibitor Ly 2940002 and then 20 ng/ml of TNF-α for 72 h. **(C)**. Cell apoptosis was determined by TUNEL assay in samples treated as in (A) and (B). **(D)**. Proteins from similarly treated cells were subjected to western blot to detect caspase 3 cleavage and key pro/anti-apoptotic molecules.

Aberrant NF-κB activity in cancer cells is implicated in apoptosis resistance to various stimuli, including inflammatory cytokines like TNF-α [17]. Activated Akt was implicated in proliferation/survival of PC cells and/or resistance to TNF-α mediated cell death in various cancers [29,30]. Here, we found that MSLN-induced NF-κB rescues cells from TNF-α-induced apoptosis. Pre-treatment with the pharmacological IKK inhibitor wedelolactone or the PI3K inhibitor Ly2940002 rendered MIA-MSLN cells sensitive to TNF-α-mediated viability reduction (Figure 6A, B) and apoptosis (Figure 6C). While the MIA-V cells had a 45% reduction in cell viability following TNF-α treatment, 100% of MIA-MSLN cells remained viable after 3 days of treatment. Twelve hours pre-treatment with Wedelactone, however, made MIA-MSLN cells extremely sensitive to TNF-α-induced apoptosis, with 83% and 93% reduction in viability respectively with 12.5 μg/ml and 25 μg/ml of Wedelolactone (Figure 6A). Pretreatment with Ly294002 also reduced viability to similar levels (Figure 6B). The TUNEL-positive apoptotic fraction of MIA-MSLN cells after TNF-α treatment (20 ng/ml, 72 h) increased from 5% to 80% (Figure 6C) upon pre-treatment with 25 μg/ml of Wedelolactone and 44% upon pretreatment with Ly294002. These data clearly indicate a role of constitutive NF-κB as well as activated Akt in MIA-MSLN cells' protection from TNF-α-induced apoptosis.

### Inhibiting PI3K and NF-κB activity increases Caspase activity and decreases anti-apoptotic protein expression in MIA-MSLN cells

We further found that both Ly294002 & Wedelolactone pre-treatment increased caspase-3 activation in MIA-MSLN cells after TNF-α treatment compared with controls (Figure 6D). In addition, the levels of Mcl-1/Bcl-XL were also reduced in Wedelolactone/TNF-α-treated MIA-MSLN (Figure 6D). Interestingly, we did not find a reduction in Mcl-1 by Ly294002 pre-treated cells. We then did a cell cycle analysis (Additional File 4, Fig. S4) with similar treatments and found that Ly2940002/TNF-α caused a G1 arrest (from 54.6%G1/26.6%S to 73.6%G1/12.4%S) of MIA-MSLN. In contrast, Wedelolactone/TNF-α treatment decreased S phase cells (to 17.4%) but increased G2 (23.9%) and partly increased sub G0 cells thus denoting a G2 arrest and apoptosis. Our data strongly implicate Mcl-1 may act as a major molecule that protecting MSLN overexpressing cells from TNF-α-mediated cytotoxicity.

### Silencing the endogenous production of IL-6 in MIA-MSLN cells increases the sensitivity of the cells to TNF-α-mediated cytotoxicity

**Figure 7:**
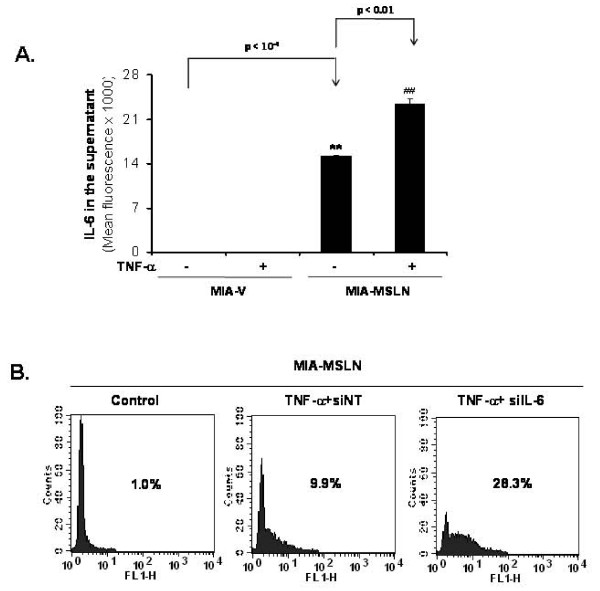
**High levels of IL-6 in MIA-MSLN cells plays important role in resistance to TNF-α-mediated cytotoxicity**. **(A)**. Elevated constitutive and TNF-α induced IL-6 expression in MIA-MSLN cells using Luminex-based IL-6 assay kit. Y-axis represents fluorescence corresponding to IL-6-levels. Bars denote SD of duplicate data. **(B)**. Inhibiting endogenous IL-6 by siRNA sensitizes MIA-MSLN cells to TNF-α-induced apoptosis. MIA-MSLN cells were either mock-transfected (control) or transfected with non-targeting (siNT) or IL-6 specific siRNA pools (siIL-6) and treated with 20 ng/ml of TNF-α for 72 h and analyzed for TUNEL positivity. Flow histograms show percentage of dUTP-FITC-positive apoptotic cells. Plots are representatives of three independent experiments.

We previously showed that cells overexpressing MSLN either naturally (e.g. BxPC3) or artificially (e.g. MIA-MSLN) express increased IL-6 at both mRNA/protein levels [31]. We also found this IL-6 was NF-κB-regulated. Previous studies showed IL-6 as protective against cytokine-induced apoptosis in various cells [32,33]. We asked whether protective effect of MSLN against TNF-α is due to increased IL-6. We examined IL-6 production by MIA-V/MIA-MSLN cells ± TNF-α treatment. As demonstrated in Figure 7A, a significantly higher production of IL-6 was observed in MIA-MSLN cells, and this was further increased by TNF-α treatment. We then blocked IL-6 production using IL-6 specific siRNA pool and examined the effect of TNF-α treatment on apoptosis by TUNEL. Shown in Figure 7B, IL-6-silenced MIA-MSLN cells had increased apoptotic (28% TUNEL positive) fraction after TNF-α treatment compared to only 9% in non-targeting siRNA transfected cells, indicating the protective role of IL-6.

## Discussion

In our previous publications, we have reported that MSLN overexpression is associated with increased migration [3], and increased colony forming ability under both anchorage independence as well as in matrigel [31]. These characteristics all pointing towards a tumorigenic role and metastasis related phenotypes of mesothelin. The current study is a significant step forward in understanding the role of MSLN in PC pathogenesis, especially with regards to cancer cell survival in a highly inflammatory milieu, a hallmark of this deadly cancer. There is a clear correlation between MSLN level and resistance to TNF-α induced apoptosis in various PC cells lines. We also convincingly show that forced overexpression of MSLN in the PC cell line MIA PaCa-2 made these cells resistant to TNF-α-induced growth inhibition/apoptosis, and that silencing endogenous MSLN in AsPC-1 cells renders these cells sensitive. TNF-α also induced S-phase promoting effects in MIA-MSLN cells with concomitant induction of cyclin A. Furthermore, an increased constitutive activation of NF-κB in MIA-MSLN cells seemed to be responsible for its apparent TNF-α resistance, as blocking this activation using the IKK inhibitor wedelolactone significantly increased the sensitivity to TNF-α-mediated cytotoxicity. Similarly, an activated Akt pathway also contributes towards resistance to growth inhibition by TNF-α. Furthermore, high endogenous production of NF-κB-regulated IL-6 and increased Mcl-1 seem to be direct players in this protection. Our results describe a novel function for MSLN, inducing an NF-κB/Akt-dependent, anti-apoptotic pathway which can protect PC cells from TNF-α-induced apoptosis, and describe a probable mechanism of PC cell survival in midst of inflammation and inflammatory mediators.

Previous data [14,34] and ours presented here show that PC cell lines Capan-1, BxPC3, PL 45, Hs 766T, AsPC-1, Capan-2, Panc 48 cells are resistant to TNF-α. Interestingly, all these cells have high MSLN expression [35], while TNF-α sensitive cells such as MIA PaCa-2 and Panc 28 expresses low MSLN [35]. In addition, silencing MSLN expression in AsPc-1/MIA-MSLN cells reversed the TNF-α resistance, indicating a direct role of MSLN. The ability of normal mesothelial and mesothelioma cells [36-38] to survive/proliferate in presence of TNF-α is intriguing. Another interesting observation is that TNF-α induces MIA-MSLN cells to go into cell cycle rather than stalling the process, similar to previous reports of auto/paracrine growth stimulation by TNF-α [17]. PC cells are prone to proliferation by TNF-α through up-regulation of PDGF [39] and/or EGFR/TGF-α [19]. Given our data it's a strong proposition that MSLN contribute towards this. Further, our PI3 kinase inhibition data indicates that the cells' ability to evade growth inhibition might be due to activated Akt found in these cells.

Mesothelin promotes both survival and proliferation under anchorage dependence [1,3] and independence [4,31] in low serum conditions [31]. Furthermore, loss of cyclin A and G1-cell cycle seemed to proceed cell-killing by TNF-α in endothelial cells & TNF-α promoted cell cycle progression and cyclin A synthesis [26,27]. Based on these facts, we raised a question whether a lack of cell cycle promotion by TNF-α is essential for its pro-apoptotic role in MSLN-low cells. In addition, caspase inhibition not only decreased the apoptotic sub-G0 fraction (from 8% to 2.9%), but also increased MIA-V cells in S phase (from 15.4% to 26.9%) by TNF-α treatment (Figure 4C). This indicates that a failure to go into DNA synthesis is linked to MIA-V cells' induction to apoptosis by TNF-a treatment.

Bcl-2 regulates apoptosis by heterodimerization with its pro-apoptotic partner, Bax, to maintain mitochondrial integrity [40]. Phosphorylation of Bcl-2 at Ser70 is necessary for its anti-apoptotic function [41]. Although we have not identified the responsible kinase, nevertheless an increased p-Ser70 in MIA-MSLN cells is a significant observation with respect to its anti-apoptotic role. BAD is a pro-apoptotic Bcl-2 family member coordinating survival and mitochondrial cell death signals [42]. BAD phosphorylation is essential to block apoptosis [24]. An increased Ser75 phosphorylation of BAD in MIA-MSLN cells could be thus critical for its anti-apoptotic functions. PI3K activity supports EGF/serum induced survival of cancer cells, through phosphorylation-mediated inactivation of BAD [43] although other kinases could also control Ser75 phosphorylation [25]. Given that inhibiting PI3K activity rendered MIA-MSLN cells sensitive to TNF-α, the pathway could be involved in BAD-inactivation and hence protection from TNF-α. Another interesting finding is that Mcl-1 is a major player in MSLN-induced resistance to TNF-α. Although primarily a Stat3 regulated gene, NF-κB regulated Mcl-1 is responsible for TRAIL resistance in various tumors [44,45] including the MSLN-high PC cell line BxPC-3 [46]. The fact that MIA-MSLN cells have activated Stat3 [1] indicates a probable role of the factor in TNF-α resistance as well. MSLN-induced Mcl-1 upregulation has been reported in taxol resistant ovarian cancer cells [47] indicating its importance in MSLN induced protection from various apoptotic stimuli.

TNF-α induces NF-κB activation in most cells [17,18] but this is not always sufficient to avoid the pro-apoptotic effects of TNF-α; rather, a constitutive activation of NF-κB seems to be necessary [20]. Similarly, we found that constitutively activated NF-κB is responsible for resistance to TNF-α-induced apoptosis. Autocrine IL-6-induced Mcl-1 could protect prostate cancer cells from apoptosis [33]. IL-6 also protects pancreatic islet beta cells from TNF-α-induced cell death [32]. Incidentally, MSLN is highly expressed in the islet beta cells of developing rat pancreas [48] leading one to speculate its role in IL-6 production and subsequent TNF-α protection. We found that MIA-MSLN cells produce increased IL-6 in a NF-κB regulated manner [31] and IL-6 production increased significantly upon TNF-α treatment. In fact, studies reveal that co-operative action of IL-6 and TNF-α induces proliferation during liver regeneration after multiple partial hepatectomy [49]. Thus it could be the co-operativity between the two that results in S-phase induction by TNF-α under reduced serum conditions. The elevated IL-6 may be responsible for increased Mcl-1 expression and partially contribute to Stat3 activation in these cells. More precisely, we hypothesize that a MSLN-NF-κB-IL-6-Stat3-Mcl-1 axis may be a major survival axis operating in these cells. Our data clearly show that silencing endogenous IL-6 increased sensitivity of MIA-MSLN cells to TNF-α-induced cell death. Although Mcl-1 is not a direct target for Akt, NF-kappa B and Akt signaling pathways can converge. Therefore, an indirect relationship could be established between Akt and Mcl-1 through NF-κB. MSLN-Akt-NF-kB-IL-6-Mcl-1 survival axis could be unique for PC cells.

The recent success of intratumorally-injected adeno-encoded, radiation-inducible-promoter driven hTNF-α, AdEgr.TNF.11D (TNFerade)+gemcitabine [12] proves TNF-α could still be useful as anti-tumor agent. The balance between cytotoxicity and stimulation of growth factor synthesis determines which biological effects will finally result from TNF-α. This critical balance eventually decides which PC cells might escape the anti-proliferative effects of TNF-α, and the effects that these properties will have on patient tumors. Deciphering the mechanism of TNF-α resistance in MSLN-expressing tumors would thus help provide rationale for designing combined chemotherapy with TNF-α ± MSLN inhibition to efficiently target these tumors.

## Conclusions

In summary, we found that MSLN overexpressing cells are generally resistant to TNF-α induced cytotoxicity and cell inhibition, which helps the cells thrive in an inflammatory milieu which is a hallmark of PC. There is a renewed interest in intratumorally-injected TNFerade in conjunction with conventional chemotherapy in PC without metastasis at diagnosis. Because MSLN is expressed in about 90% of PC patients, a simultaneous blocking of its action is warranted for the success of a therapeutic regimen.

## List of abbreviations

MSLN: mesothelin; PC: pancreatic cancer; TNF-α: Tumor necrosis factor-*α*; TNFR: tumor necrosis factor receptor; TNFerade: adeno-encoded, chemo/radiation-inducible-promoter driven hTNF-α; MIA-MSLN: a MIA PaCa2 cell line that stably overexpressing MSLN; MIA-V: a MIA PaCa2 cell line with vector control; MIA-GFP: a MIA PaCa2 cell line that stably overexpressing GFP; HPDE: Human pancreatic ductal epithelial cell

## Competing interests

The authors declare that they have no competing interests.

## Authors' contributions

UB planned and performed most experiments, wrote the manuscript. CMM helped in many of these experiments and helped in making the plasmid preparations as well helped in editing the manuscript. ML and CC participated in discussion. QY participated in discussion and manuscript preparation and provided grant support for this study. All authors read and approved the final version of this manuscript.

## Supplementary Material

Additional file 1 **Additional Figure S1: Effect of serum on the resistance to TNF-a mediated cytotoxicity**. The MSLN-high BxPC3 and AsPC-1 cells were tested for viability by MTT assay after treatment with 20 ng/ml of TNF-a for 72 h. Data plotted shown mean value from quadruplicate wells. Click here for file

Additional file 2 **Additional Figure S2: Knocking-down MSLN expression in MIA-MSLN cells by specific siRNA directed against MSLN**. Y axis shows the GAPDH-normalized mRNA levels as 2^[Ct(GAPDH)-Ct(MSLN)]. Bars denote s.d. of duplicate data. *, # denote p < 0.05, **, ## denote p < 0.01, compared with controls by using t test. Click here for file

Additional file 3 **Additional Figure S3: TNFR1 expression levels in MIA-V/MIA-MSLN cells**. (A). Real-time PCR analysis of TNFR1 relative mRNA levels. Results denote GAPDH-normalized mRNA levels as 2^[Ct(GAPDH)-Ct(TNFR1)]. The bars denote s.d. of duplicate data. (B). Western blot data showing TNFR1 protein expression in MIA-V/MIA-MSLN cells. Click here for file

Additional file 4 **Additional Figure S4: Sub-confluent MIA-V/MIA-MSLN cells were serum starved for 24 h and then treated with 10/20 ng/ml of TNF-α for 72 h**. Cells were collected and fixed, PI-stained, and analyzed for cell cycle phase distribution (percentage of cells) with FACS. Percentage of cells in G0/G1, S, G2/M phase are shown against the respective peaks in the histograms. Click here for file

## References

[B1] Bharadwaj U Li M Chen C Yao Q Mesothelin-induced pancreatic cancer cell proliferation involves alteration of cyclin E via activation of signal transducer and activator of transcription protein 3 Mol Cancer Res 2008 6 1755 1765 10.1158/1541-7786.MCR-08-0095 19010822 PMC2929833

[B2] Hassan R Ho M Mesothelin targeted cancer immunotherapy Eur J Cancer 2008 44 46 53 10.1016/j.ejca.2007.08.028 17945478 PMC2265108

[B3] Li M Bharadwaj U Zhang R Zhang S Mu H Fisher WE Brunicardi FC Chen C Yao Q Mesothelin is a malignant factor and therapeutic vaccine target for pancreatic cancer Mol Cancer Ther 2008 7 286 296 10.1158/1535-7163.MCT-07-0483 18281514 PMC2929838

[B4] Uehara N Matsuoka Y Tsubura A Mesothelin promotes anchorage-independent growth and prevents anoikis via extracellular signal-regulated kinase signaling pathway in human breast cancer cells Mol Cancer Res 2008 6 186 193 10.1158/1541-7786.MCR-07-0254 18245228

[B5] Cheng WF Huang CY Chang MC Hu YH Chiang YC Chen YL Hsieh CY Chen CA High mesothelin correlates with chemoresistance and poor survival in epithelial ovarian carcinoma Br J Cancer 2009 100 1144 1153 10.1038/sj.bjc.6604964 19293794 PMC2669998

[B6] Koorstra JB Hustinx SR Offerhaus GJ Maitra A Pancreatic carcinogenesis Pancreatology 2008 8 110 125 10.1159/000123838 18382097 PMC2663569

[B7] Szlosarek P Charles KA Balkwill FR Tumour necrosis factor-alpha as a tumour promoter Eur J Cancer 2006 42 745 750 10.1016/j.ejca.2006.01.012 16517151

[B8] Balkwill F TNF-alpha in promotion and progression of cancer Cancer Metastasis Rev 2006 25 409 416 10.1007/s10555-006-9005-3 16951987

[B9] Carswell EA Old LJ Kassel RL Green S Fiore N Williamson B An endotoxin-induced serum factor that causes necrosis of tumors Proc Natl Acad Sci USA 1975 72 3666 3670 10.1073/pnas.72.9.3666 1103152 PMC433057

[B10] Fernandez A Marin MC McDonnell T Ananthaswamy HN Differential sensitivity of normal and Ha-ras-transformed C3H mouse embryo fibroblasts to tumor necrosis factor: induction of bcl-2, c-myc, and manganese superoxide dismutase in resistant cells Oncogene 1994 9 2009 2017 8208546

[B11] Komori A Yatsunami J Suganuma M Okabe S Abe S Sakai A Sasaki K Fujiki H Tumor necrosis factor acts as a tumor promoter in BALB/3T3 cell transformation Cancer Res 1993 53 1982 1985 8481899

[B12] Murugesan SR King CR Osborn R Fairweather WR O'Reilly EM Thornton MO Wei LL Combination of human tumor necrosis factor-alpha (hTNF-alpha) gene delivery with gemcitabine is effective in models of pancreatic cancer Cancer Gene Ther 2009 10.1038/cgt.2009.32 19444305

[B13] Weichselbaum RR Kufe D Translation of the radio- and chemo-inducible TNFerade vector to the treatment of human cancers Cancer Gene Ther 2009 16 609 619 10.1038/cgt.2009.37 19444302

[B14] Lyu MA Kurzrock R Rosenblum MG The immunocytokine scFv23/TNF targeting HER-2/neu induces synergistic cytotoxic effects with 5-fluorouracil in TNF-resistant pancreatic cancer cell lines Biochem Pharmacol 2008 75 836 846 10.1016/j.bcp.2007.10.013 18082672

[B15] Wang CY Cusack EV Jr Liu R Baldwin EV Jr Control of inducible chemoresistance: enhanced anti-tumor therapy through increased apoptosis by inhibition of NF-kappaB Nat Med 1999 5 412 417 10.1038/7410 10202930

[B16] Schmiegel WH Caesar J Kalthoff H Greten H Schreiber HW Thiele HG Antiproliferative effects exerted by recombinant human tumor necrosis factor-alpha (TNF-alpha) and interferon-gamma (IFN-gamma) on human pancreatic tumor cell lines Pancreas 1988 3 180 188 10.1097/00006676-198804000-00012 3131759

[B17] Aggarwal BB Shishodia S Sandur SK Pandey MK Sethi G Inflammation and cancer: how hot is the link? Biochem Pharmacol 2006 72 1605 1621 10.1016/j.bcp.2006.06.029 16889756

[B18] Aggarwal BB Signalling pathways of the TNF superfamily: a double-edged sword Nat Rev Immunol 2003 3 745 756 10.1038/nri1184 12949498

[B19] Schmiegel W Roeder C Schmielau J Rodeck U Kalthoff H Tumor necrosis factor alpha induces the expression of transforming growth factor alpha and the epidermal growth factor receptor in human pancreatic cancer cells Proc Natl Acad Sci USA 1993 90 863 867 10.1073/pnas.90.3.863 8430098 PMC45770

[B20] Giri DK Aggarwal BB Constitutive activation of NF-kappaB causes resistance to apoptosis in human cutaneous T cell lymphoma HuT-78 cells. Autocrine role of tumor necrosis factor and reactive oxygen intermediates J Biol Chem 1998 273 14008 14014 10.1074/jbc.273.22.14008 9593751

[B21] Deng X Kornblau SM Ruvolo PP May EV Jr Regulation of Bcl2 phosphorylation and potential significance for leukemic cell chemoresistance J Natl Cancer Inst Monogr 2001 30 37 10.1093/oxfordjournals.jncimonographs.a024254 11158204

[B22] Cory S Adams JM The Bcl2 family: regulators of the cellular life-or-death switch Nat Rev Cancer 2002 2 647 656 10.1038/nrc883 12209154

[B23] Downward J How BAD phosphorylation is good for survival Nat Cell Biol 1999 1 E33 35 10.1038/10026 10559890

[B24] Datta SR Ranger AM Lin MZ Sturgill JF Ma YC Cowan CW Dikkes P Korsmeyer SJ Greenberg ME Survival factor-mediated BAD phosphorylation raises the mitochondrial threshold for apoptosis Dev Cell 2002 3 631 643 10.1016/S1534-5807(02)00326-X 12431371

[B25] Eisenmann KM VanBrocklin MW Staffend NA Kitchen SM Koo HM Mitogen-activated protein kinase pathway-dependent tumor-specific survival signaling in melanoma cells through inactivation of the proapoptotic protein bad Cancer Res 2003 63 8330 8337 14678993

[B26] Spyridopoulos I Mayer P Shook KS Axel DI Viebahn R Karsch KR Loss of cyclin A and G1-cell cycle arrest are a prerequisite of ceramide-induced toxicity in human arterial endothelial cells Cardiovasc Res 2001 50 97 107 10.1016/S0008-6363(01)00196-1 11282082

[B27] Volland S Amtmann E Sauer G TNF accelerates the S-phase of the cell cycle in tumor cells Int J Cancer 1994 56 698 705 10.1002/ijc.2910560515 8314347

[B28] Satyanarayana A Kaldis P Mammalian cell-cycle regulation: several Cdks, numerous cyclins and diverse compensatory mechanisms Oncogene 2009 28 2925 2939 10.1038/onc.2009.170 19561645

[B29] Liu CJ Lo JF Kuo CH Chu CH Chen LM Tsai FJ Tsai CH Tzang BS Kuo WW Huang CY Akt Mediates 17 beta-Estradiol and/or Estrogen Receptor alpha Inhibition of LPS-Induced Tumor Necrosis Factor-alpha Expression and Myocardial Cell Apoptosis by Suppressing the JNK1/2-NFkappaB Pathway J Cell Mol Med 2009 10.1111/j.1582-4934.2009.00669.x PMC4516514 20196785

[B30] Qiu J Xiao J Han C Li N Shen X Jiang H Cao X Potentiation of tumor necrosis factor-alpha-induced tumor cell apoptosis by a small molecule inhibitor for anti-apoptotic protein hPEBP4 J Biol Chem 285 12241 12247 10.1074/jbc.M110.111898 PMC2852963 20177075

[B31] Bharadwaj U Marin-Muller C Li M Chen C Yao Q Mesothelin Overexpression Promotes Autocrine IL-6/sIL-6R Trans-signaling to stimulate Pancreatic Cancer Cell Proliferation Carcinogenesis 2011 32 1013 1024 10.1093/carcin/bgr075 21515913 PMC3128561

[B32] Choi SE Choi KM Yoon IH Shin JY Kim JS Park WY Han DJ Kim SC Ahn C Kim JY IL-6 protects pancreatic islet beta cells from pro-inflammatory cytokines-induced cell death and functional impairment in vitro and in vivo Transpl Immunol 2004 13 43 53 10.1016/j.trim.2004.04.001 15203128

[B33] Cavarretta IT Neuwirt H Untergasser G Moser PL Zaki MH Steiner H Rumpold H Fuchs D Hobisch A Nemeth JA Culig Z The antiapoptotic effect of IL-6 autocrine loop in a cellular model of advanced prostate cancer is mediated by Mcl-1 Oncogene 2007 26 2822 2832 10.1038/sj.onc.1210097 17072336

[B34] Bai J Sui J Demirjian A Vollmer EV Jr Marasco W Callery MP Predominant Bcl-XL knockdown disables antiapoptotic mechanisms: tumor necrosis factor-related apoptosis-inducing ligand-based triple chemotherapy overcomes chemoresistance in pancreatic cancer cells in vitro Cancer Res 2005 65 2344 2352 10.1158/0008-5472.CAN-04-3502 15781649

[B35] Argani P Iacobuzio-Donahue C Ryu B Rosty C Goggins M Wilentz RE Murugesan SR Leach SD Jaffee E Yeo CJ Mesothelin is overexpressed in the vast majority of ductal adenocarcinomas of the pancreas: identification of a new pancreatic cancer marker by serial analysis of gene expression (SAGE) Clin Cancer Res 2001 7 3862 3868 11751476

[B36] Catalan MP Subira D Reyero A Selgas R Ortiz-Gonzalez A Egido J Ortiz A Regulation of apoptosis by lethal cytokines in human mesothelial cells Kidney Int 2003 64 321 330 10.1046/j.1523-1755.2003.00062.x 12787425

[B37] Gordon GJ Mani M Mukhopadhyay L Dong L Yeap BY Sugarbaker DJ Bueno R Inhibitor of apoptosis proteins are regulated by tumour necrosis factor-alpha in malignant pleural mesothelioma J Pathol 2007 211 439 446 10.1002/path.2120 17253597

[B38] Yang H Bocchetta M Kroczynska B Elmishad AG Chen Y Liu Z Bubici C Mossman BT Pass HI Testa JR TNF-alpha inhibits asbestos-induced cytotoxicity via a NF-kappaB-dependent pathway, a possible mechanism for asbestos-induced oncogenesis Proc Natl Acad Sci USA 2006 103 10397 10402 10.1073/pnas.0604008103 16798876 PMC1502469

[B39] Kalthoff H Roeder C Humburg I Thiele HG Greten H Schmiegel W Modulation of platelet-derived growth factor A- and B-chain/c-sis mRNA by tumor necrosis factor and other agents in adenocarcinoma cells Oncogene 1991 6 1015 1021 1906154

[B40] Yip KW Reed JC Bcl-2 family proteins and cancer Oncogene 2008 27 6398 6406 10.1038/onc.2008.307 18955968

[B41] Deng X Ruvolo P Carr B May EV Jr Survival function of ERK1/2 as IL-3-activated, staurosporine-resistant Bcl2 kinases Proc Natl Acad Sci USA 2000 97 1578 1583 10.1073/pnas.97.4.1578 10677502 PMC26477

[B42] Bergmann A Survival signaling goes BAD Dev Cell 2002 3 607 608 10.1016/S1534-5807(02)00328-3 12431365

[B43] Chao OS Clement MV Epidermal growth factor and serum activate distinct pathways to inhibit the BH3 only protein BAD in prostate carcinoma LNCaP cells Oncogene 2006 25 4458 4469 10.1038/sj.onc.1209421 16767165

[B44] Ricci MS Kim SH Ogi K Plastaras JP Ling J Wang W Jin Z Liu YY Dicker DT Chiao PJ Reduction of TRAIL-induced Mcl-1 and cIAP2 by c-Myc or sorafenib sensitizes resistant human cancer cells to TRAIL-induced death Cancer Cell 2007 12 66 80 10.1016/j.ccr.2007.05.006 17613437

[B45] Wang X Chen W Zeng W Bai L Tesfaigzi Y Belinsky SA Lin Y Akt-mediated eminent expression of c-FLIP and Mcl-1 confers acquired resistance to TRAIL-induced cytotoxicity to lung cancer cells Mol Cancer Ther 2008 7 1156 1163 10.1158/1535-7163.MCT-07-2183 18483303 PMC2715176

[B46] Huang S Sinicrope FA BH3 mimetic ABT-737 potentiates TRAIL-mediated apoptotic signaling by unsequestering Bim and Bak in human pancreatic cancer cells Cancer Res 2008 68 2944 2951 10.1158/0008-5472.CAN-07-2508 18413764 PMC2948486

[B47] Chang MC Chen CA Hsieh CY Lee CN Su YN Hu YH Cheng WF Mesothelin inhibits paclitaxel-induced apoptosis through the PI3K pathway Biochem J 2009 424 449 458 10.1042/BJ20082196 19747165

[B48] Hou LQ Wang YH Liu LJ Guo J Teng LP Cao LH Shi H Yuan L De W Expression and localization of mesothelin in developing rat pancreas Dev Growth Differ 2008 50 531 541 10.1111/j.1440-169X.2008.01047.x 18505465

[B49] Ledda-Columbano GM Curto M Piga R Zedda AI Menegazzi M Sartori C Shinozuka H Bluethmann H Poli V Ciliberto G Columbano A In vivo hepatocyte proliferation is inducible through a TNF and IL-6-independent pathway Oncogene 1998 17 1039 1044 10.1038/sj.onc.1202018 9747883

