# Supplementary figures and images for: Mesothelin confers pancreatic cancer cell resistance to TNF-α-induced apoptosis through Akt/PI3K/NF-κB activation and IL-6/Mcl-1 overexpression
Source: Mol Cancer. 2011 Aug 31;10:106. doi: 10.1186/1476-4598-10-106 (PMC3175472; doi:10.1186/1476-4598-10-106)

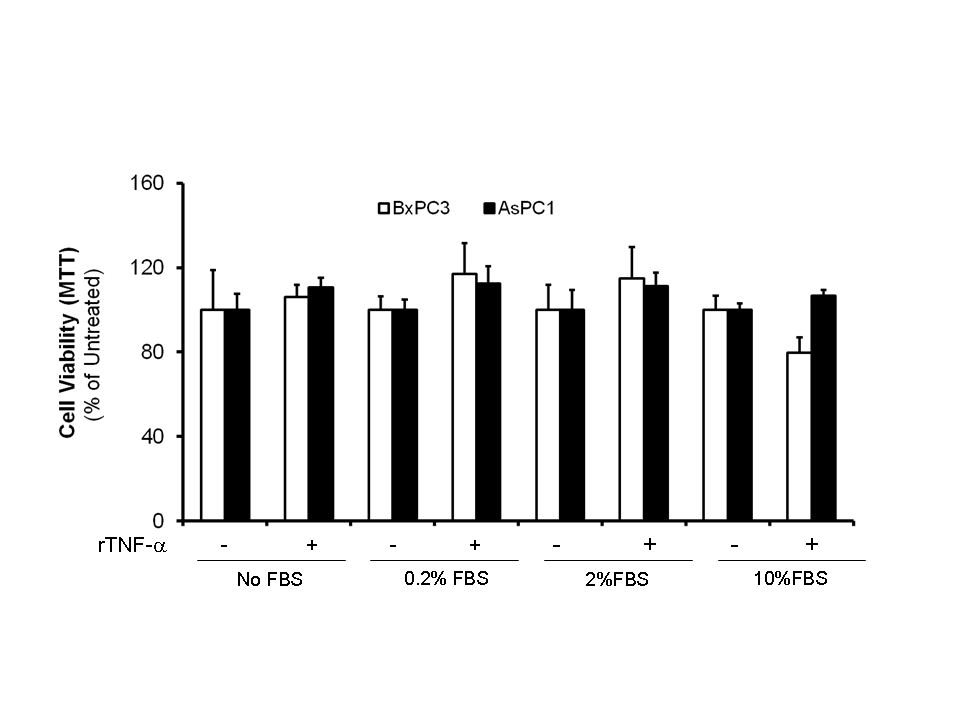

Supplement: Additional file 1 — Additional Figure S1: Effect of serum on the resistance to TNF-a mediated cytotoxicity. The MSLN-high BxPC3 and AsPC-1 cells were tested for viability by MTT assay after treatment with 20 ng/ml of TNF-a for 72 h. Data plotted shown mean value from quadruplicate wells. [file 1476-4598-10-106-S1.TIFF]

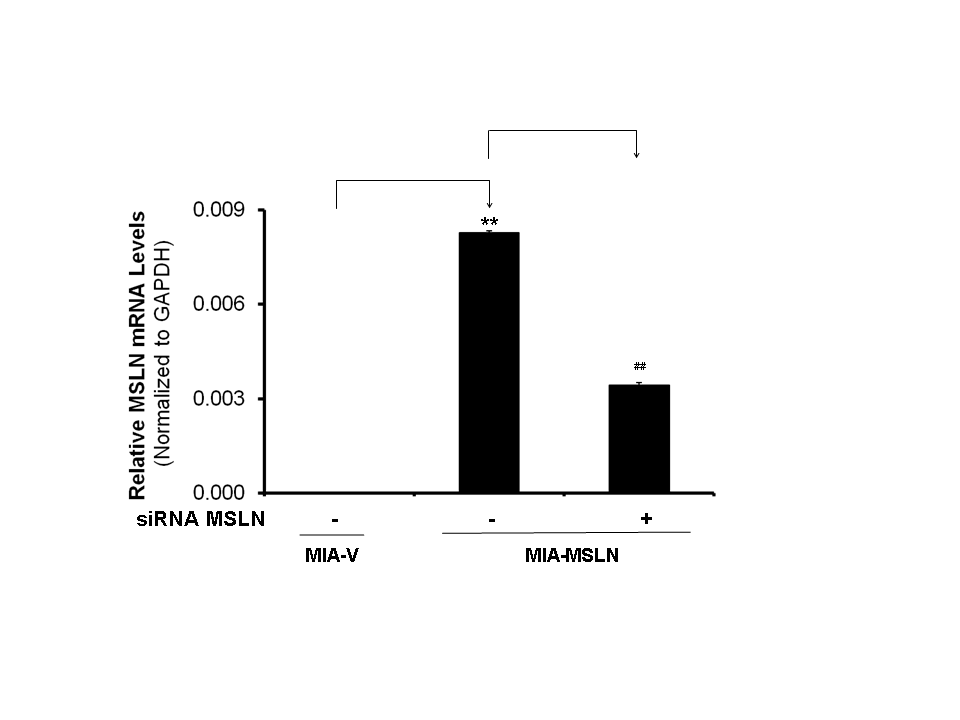

Supplement: Additional file 2 — Additional Figure S2: Knocking-down MSLN expression in MIA-MSLN cells by specific siRNA directed against MSLN. Y axis shows the GAPDH-normalized mRNA levels as 2^[Ct(GAPDH)-Ct(MSLN)]. Bars denote s.d. of duplicate data. *, # denote p < 0.05, **, ## denote p < 0.01, compared with controls by using t test. [file 1476-4598-10-106-S2.TIFF]

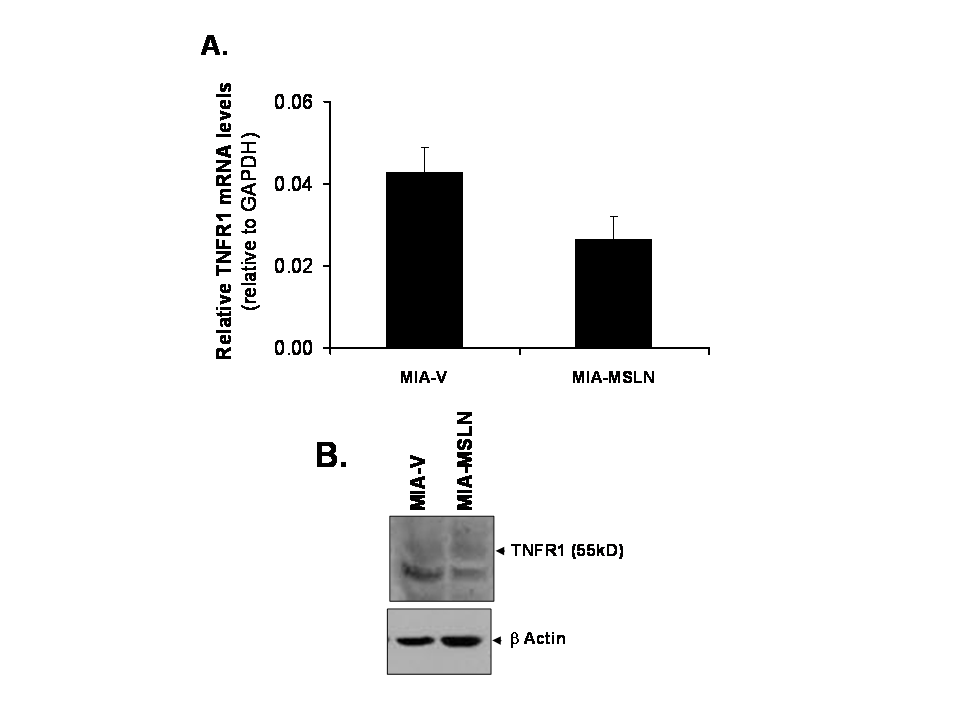

Supplement: Additional file 3 — Additional Figure S3: TNFR1 expression levels in MIA-V/MIA-MSLN cells. (A). Real-time PCR analysis of TNFR1 relative mRNA levels. Results denote GAPDH-normalized mRNA levels as 2^[Ct(GAPDH)-Ct(TNFR1)]. The bars denote s.d. of duplicate data. (B). Western blot data showing TNFR1 protein expression in MIA-V/MIA-MSLN cells. [file 1476-4598-10-106-S3.TIFF]

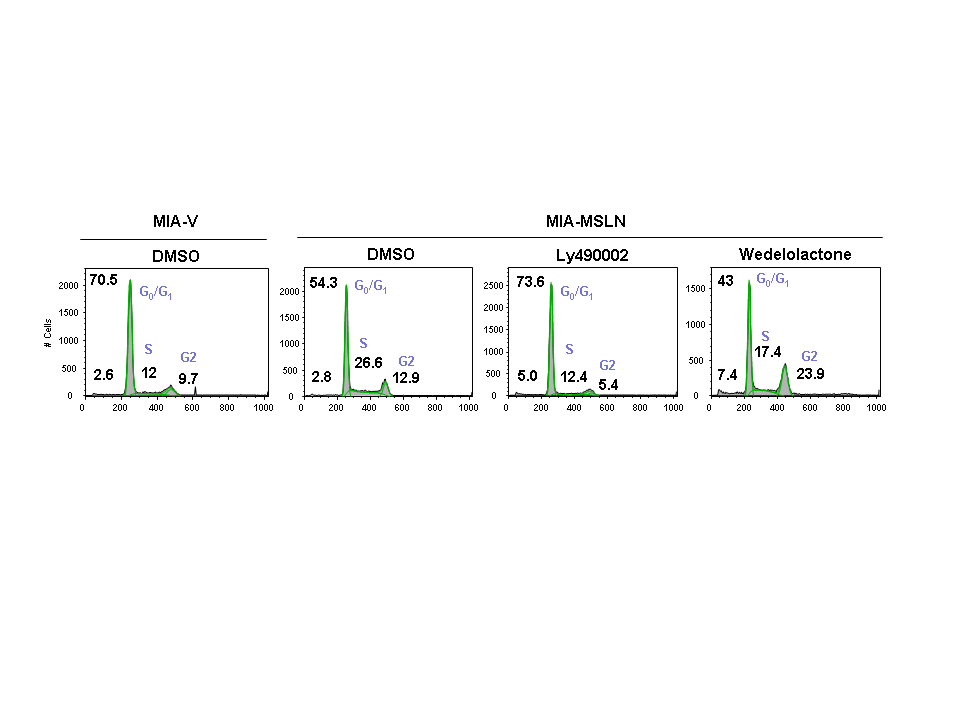

Supplement: Additional file 4 — Additional Figure S4: Sub-confluent MIA-V/MIA-MSLN cells were serum starved for 24 h and then treated with 10/20 ng/ml of TNF-α for 72 h. Cells were collected and fixed, PI-stained, and analyzed for cell cycle phase distribution (percentage of cells) with FACS. Percentage of cells in G0/G1, S, G2/M phase are shown against the respective peaks in the histograms. [file 1476-4598-10-106-S4.TIFF]
